# Teledentistry for Homebound and Institutionalized Older Adults: A Rapid Review for Its Potential Use in Primary Healthcare

**DOI:** 10.1002/puh2.70297

**Published:** 2026-06-12

**Authors:** Gabriel Schmitt da Cruz, Elaine Caroline Ferreira, Gabriela Bampi, Eduardo Dickie de Castilhos, Maria Inês Meurer, Jose Antonio Gil‐Montoya, Ana Lúcia Schaefer Ferreira de Mello

**Affiliations:** ^1^ Graduate Program in Dentistry Federal University of Santa Catarina Florianópolis Brazil; ^2^ Dentistry Undergraduate Course Federal University of Santa Catarina Florianópolis Brazil; ^3^ Graduate Program in Dentistry Federal University of Pelotas Pelotas Brazil; ^4^ Pathology Department Federal University of Santa Catarina Florianópolis Brazil; ^5^ Faculty of Dentistry University of Granada Granada Spain

**Keywords:** aged, dental care for aged, homebound persons, teledentistry

## Abstract

**Objective:**

To investigate teledentistry practices for oral healthcare of homebound and institutionalized older adults and to discuss their potential applicability to primary healthcare (PHC) settings.

**Method:**

A rapid literature review, structured by the SPICE model, searched 10 databases in October 2024, without language or date restrictions. Two independent researchers screened the studies, and a third researcher resolved disagreements. Data were tabulated to capture study characteristics, teledentistry intervention features, and reported outcomes. The study was registered in the Open Science Framework (OSF) (DOI: 10.17605/OSF.IO/FD3PN).

**Results:**

Sixteen studies were included, comprising 15 teledentistry interventions targeting homebound or institutionalized older adults (≥60 years), conducted primarily in the United States, Japan, Australia, and Chile. Interventions took place at home (*n* = 9) or in long‐term care institutions (*n* = 7), with sample sizes ranging from 4 to 252 participants and a mean duration of 6.7 months. No intervention was implemented within PHC settings. Applications included oral health assessment, treatment planning and referral, oral health education, and rehabilitation of oral function. Technological approaches were predominantly asynchronous, followed by synchronous and hybrid models. Findings suggest that teledentistry may support the detection of oral conditions, identification of gingival inflammation, improvements in swallowing function, and high levels of patient satisfaction. Challenges included technical barriers and low‐digital literacy. Interprofessional collaboration with nurses and oral health technicians was frequently reported.

**Conclusions:**

Teledentistry may improve access to oral healthcare and support patient‐centered care for homebound and institutionalized older adults. However, as the included studies were not conducted within PHC settings, these findings should be interpreted as evidence of potential applicability rather than direct implementation. Further research is needed to evaluate the feasibility, effectiveness, and integration of teledentistry within PHC services.

## 1 Introduction

Population aging is a global phenomenon that poses a significant challenge to health systems worldwide [1]. The world population aged 60 years and older is projected to reach 2.1 billion by 2050, underscoring the urgent need to adapt healthcare services, including oral healthcare, to this growing demographic [1, 2]. Older adults are disproportionately affected by oral health issues, such as dental caries, periodontal disease, tooth loss, and oral cancer [3], conditions that impair basic functions like eating, speaking, and socializing, with a direct impact on overall health and quality of life [4].

Despite the importance of maintaining oral health, older adults, particularly those who are homebound or institutionalized, face considerable barriers to accessing dental care. These challenges include physical and cognitive impairments, chronic health conditions, dependency on caregivers, and reduced mobility [3]. Socioeconomic factors, such as limited financial resources and geographic isolation, further exacerbate access difficulties, resulting in unmet dental needs and worsening oral health outcomes [4, 5, 6].

Teledentistry has emerged as an innovative model of care designed to overcome these barriers, leveraging information and communication technologies to provide remote dental services [7, 8]. Recently, teledentistry has been defined as the use of technology for remote oral healthcare delivery between patients and oral healthcare providers, or between healthcare providers [9]. This approach encompasses a wide range of applications, including remote consultations, diagnostic assessments, patient education, and treatment planning, making it a versatile tool adaptable to various contexts, such as home‐based care, rural settings, and long‐term care facilities [8]. Through teledentistry, it is expected to facilitate access to care, reduce oral health inequalities, mitigate the impact of oral diseases and treatment, and promote interprofessional collaboration [9].

Operationally, teledentistry can be delivered through synchronous (real‐time), asynchronous (store‐and‐forward), and hybrid models, facilitating direct communication among patients, caregivers, and dental professionals [10]. This model not only enhances continuity of care but also minimizes the need for travel, reduces healthcare costs, and optimizes the utilization of dental professionals’ expertise [8].

However, the successful implementation of teledentistry depends on addressing several challenges. These include limited digital literacy among older adults, data privacy and patient confidentiality concerns, and the establishment of standardized protocols to ensure safe and effective remote care [11]. Furthermore, the current evidence on the clinical effectiveness of teledentistry in managing complex oral health conditions among older adults remains limited [2]. Nevertheless, existing evidence supports teledentistry as a viable strategy for improving access to oral healthcare among older adults, particularly those with limited access to conventional services [2, 3].

Despite current knowledge, it remains unclear which practices and strategies are available and reported in the literature for integrating teledentistry into primary healthcare (PHC) services. It is taken into consideration that home dental care is one of the PHC professionals’ responsibilities [12]. In addition, beyond its capacity to facilitate preventive care, early diagnosis, and timely intervention [10], teledentistry aligns with the core principles of PHC, including first‐contact access, continuity, comprehensiveness, and care coordination [13]. This alignment is particularly critical because, although oral health is considered a fundamental component of older adults’ overall health, it remains neglected, leading to a high global prevalence of preventable oral diseases [14]. Addressing this epidemic requires a strategic reform of public health policy that emphasizes prevention and surveillance and ensures the full integration of oral care into PHC services, especially to overcome the disadvantages faced by vulnerable populations [15].

According to the World Health Organization's global strategy for oral health [16], it is urgent to integrate oral health into PHC, supported by innovative workforce models, people‐centered approaches across the life course, and the optimization of digital health technologies [2].

In this context, digital health solutions, including teledentistry, should be examined for their potential to deliver accessible and effective oral healthcare. Pragmatically, there is a need to understand how dental teams and other health professionals can incorporate teledentistry into PHC models of care. However, despite growing interest in this field, it remains unclear which practices and intervention models have been described for homebound and institutionalized older adults, and how these experiences may inform their potential integration into PHC services. Importantly, existing evidence has predominantly been generated outside formal PHC settings. Despite the growing body of literature on teledentistry, there is a lack of synthesis that specifically examines how these interventions—developed predominantly outside PHC settings—may inform their potential applicability within PHC systems, particularly for homebound and institutionalized older adults.

Therefore, this rapid review aims to investigate and describe teledentistry practices for oral healthcare of homebound and institutionalized older adults and to discuss their potential applicability to PHC settings.

## 2 Methods

The rapid review followed the Joanna Briggs Institute (JBI) methodology, incorporating the SelecTing Approaches for Rapid Reviews (STARR) framework [17]. The review adhered to PRISMA‐S reporting guidelines [18], and Covidence software was used to manage and streamline the review process. The study was prospectively registered in the Open Science Framework (OSF) database in September 2024 (DOI: 10.17605/OSF.IO/FD3PN).

This rapid review was conducted using the SPICE framework [19] to structure the research question. The setting was defined as the home environment or long‐term care facilities of individuals facing barriers to accessing traditional dental services. The target population consisted of older adults with limited mobility and health conditions requiring remote dental care. The intervention under investigation was teledentistry, defined as the use of telehealth technologies for remote dental care delivery. No direct comparison was included, as the review focused on describing current practices. The evaluation component involved identifying and synthesizing literature on teledentistry protocols, guidelines, strategies, and procedures for homebound or institutionalized older adults.

On the basis of these elements, the research question guiding this review was formulated as follows: What teledentistry practices (I), their features and characteristics described in the literature (E), may inform their potential applicability to PHC (S) to serve homebound and institutionalized older adults (P), as an alternative to traditional in‐person home dental visits (C)?

This question guided the search and selection of relevant studies to ensure a comprehensive examination of teledentistry applications for this population.

Studies included in this review involved individuals aged 60 years or older, who were homebound or residing in long‐term care facilities. “Homebound” referred to individuals living in their own homes with limited mobility, whereas “institutionalized” referred to those residing in settings, such as nursing homes or retirement homes.

Studies were eligible if participants were explicitly reported as aged ≥60 years, described using equivalent terms (e.g., older adults, elderly, and geriatric populations), or if older adults were considered included. When age was not explicitly reported, studies were retained only when the population was clearly defined as older adults on the basis of explicit inclusion criteria or contextual description provided by the authors. The review included a range of study designs, including clinical trials, cross‐sectional, qualitative, and quantitative studies, to ensure a comprehensive assessment of the topic. No language or publication date restrictions were applied.

Searches were conducted in multiple databases, including PubMed, Embase, Scopus, Cochrane Library, SciELO, LILACS, CINAHL, BBO, Dentistry & Oral Sciences Source (DOSS), and Web of Science. Grey literature and narrative, scoping, and systematic reviews were excluded. Studies involving different populations, not focused on homebound or institutionalized older adults, or not addressing teledentistry, were excluded. When multiple publications described the same intervention, only the original study was considered.

The search strategy used MeSH terms and Boolean operators, with queries adapted for each database. Two independent researchers (G.S.C. and E.C.F.) screened the studies, and disagreements were resolved by a third researcher (A.L.S.F.M.). When full texts were unavailable, up to three attempts were made to contact the corresponding author via email or ResearchGate; studies with no response were excluded. The search strategy is summarized in Table 1.

**TABLE 1:** Database‐specific search strategies used in the literature review.

Database	Search strategy
**PubMed/MEDLINE**	(“Telemedicine” OR Tele* OR “Mobile Health” OR “mHealth” OR “eHealth” OR “Remote Consultation” OR Remot* OR “Teledentistry” OR “e‐dentistry”) AND (“Aged” OR “elderly” OR “older” OR “old age” OR “old aged” OR “third age” OR “Aging” OR “Senescence” OR “late‐life” OR “Geriatrics” OR Geriatric*) AND (“Mouth” OR “Mouths” OR “Oral Medicine” OR Oral* OR “Dentistry” OR “Dentists” OR Dentist* OR “Dental” OR “Tooth” OR “Teeth” OR “Stomatognathic Diseases” OR “Stomatognathic”) AND (“Homebound Persons” OR “institutionalized” OR “Home Bound” OR “Home‐Bound” OR “Shut‐Ins” OR Domicil* OR Residen* OR “Homes for the Aged” OR House* OR Home* OR Facilit*)
**Embase (Elsevier)**	(“Telemedicine” OR Tele* OR “Mobile Health” OR “mHealth” OR “eHealth” OR “Remote Consultation” OR Remot* OR “Teledentistry” OR “e‐dentistry”) AND (“Aged” OR “elderly” OR “older” OR “old age” OR “old aged” OR “third age” OR “Aging” OR “Senescence” OR “late‐life” OR “Geriatrics” OR Geriatric*) AND (“Mouth” OR “Oral Medicine” OR Oral* OR “Dentistry” OR Dentist* OR “Dental” OR “Tooth” OR “Teeth” OR “Stomatognathic”) AND (“Homebound Persons” OR institutionalized OR Domicil* OR Residen* OR “Homes for the Aged” OR Home* OR Facilit*)
**CINAHL (EBSCO)**	(“Telemedicine” OR Tele* OR “mHealth” OR “eHealth” OR “Remote Consultation” OR Remot* OR “Teledentistry”) AND (“Aged” OR elderly OR geriatric*) AND (“Oral Health” OR Dentistry OR Dentist* OR Dental) AND (“Homebound” OR institutionalized OR Domicil* OR Residen* OR “Homes for the Aged”)
**Dentistry & Oral Sciences Source (DOSS)**	(“Telemedicine” OR Tele* OR “Teledentistry” OR “e‐dentistry”) AND (“Aged” OR elderly OR geriatric*) AND (“Dentistry” OR “Oral Health”) AND (“Homebound” OR institutionalized OR Domicil* OR Residen*)
**Cochrane Library**	(“Telemedicine” OR Tele* OR “mHealth” OR “eHealth” OR “Teledentistry”) AND (“Aged” OR elderly OR geriatric*) AND (“Oral Health” OR Dentistry OR Dental) AND (“Homebound” OR institutionalized OR “Homes for the Aged”)
**Scopus (Elsevier)**	(“Telemedicine” OR Tele* OR “mHealth” OR “eHealth” OR “Teledentistry”) AND (“Aged” OR elderly OR geriatric*) AND (“Oral Health” OR Dentistry OR Dental) AND (“Homebound” OR institutionalized OR Domicil* OR Residen*)
**Web of Science (Clarivate)**	(“Telemedicine” OR Tele* OR “mHealth” OR “eHealth” OR “Teledentistry”) AND (“Aged” OR elderly OR geriatric*) AND (“Oral Health” OR Dentistry OR Dental) AND (“Homebound” OR institutionalized OR Domicil* OR Residen*)
**LILACS/BBO/BDENF**	(“Telemedicina” OR “Teleodontologia” OR “e‐odontologia” OR “eSalud” OR “Telemedicine” OR Tele* OR “mHealth” OR “eHealth” OR “Teledentistry”) AND (“Idoso” OR “Anciano” OR “Aged” OR elderly OR geriatric*) AND (“Odontologia” OR “Saúde Bucal” OR “Dentistry” OR “Oral Health”) AND (“Institucionalizado” OR “Pacientes Domiciliares” OR “Homebound” OR institutionalized OR Domicil* OR Residen*)
**SciELO**	(“Telemedicina” OR “Teleodontologia” OR “e‐odontologia” OR “Telemedicine” OR Tele* OR “Teledentistry”) AND (“Idoso” OR “Anciano” OR “Aged” OR elderly) AND (“Odontologia” OR “Saúde Bucal” OR “Dentistry” OR “Oral Health”) AND (“Institucionalizado” OR “Homebound” OR institutionalized OR Domicil* OR Residen*)

Data extraction was conducted using a structured tool, collecting key information such as authors, study title, publication year, study design, population, sample size, study objective, intervention characteristics, main findings, delivery model (synchronous/asynchronous), oral conditions assessed, professionals involved, and conclusions.

Additionally, data were tabulated, including details on first author, country, study aim, design, population characteristics, age range, intervention name, setting, number of participants, intervention duration, type of technology used, delivery model, oral conditions assessed, characteristics of the teledentistry intervention, professionals involved, indicators measured, outcomes, indices used, and study conclusions. Data were analyzed descriptively and interpreted in relation to the existing literature to provide a comprehensive synthesis of findings.

## 3 Results

**FIGURE 1:**
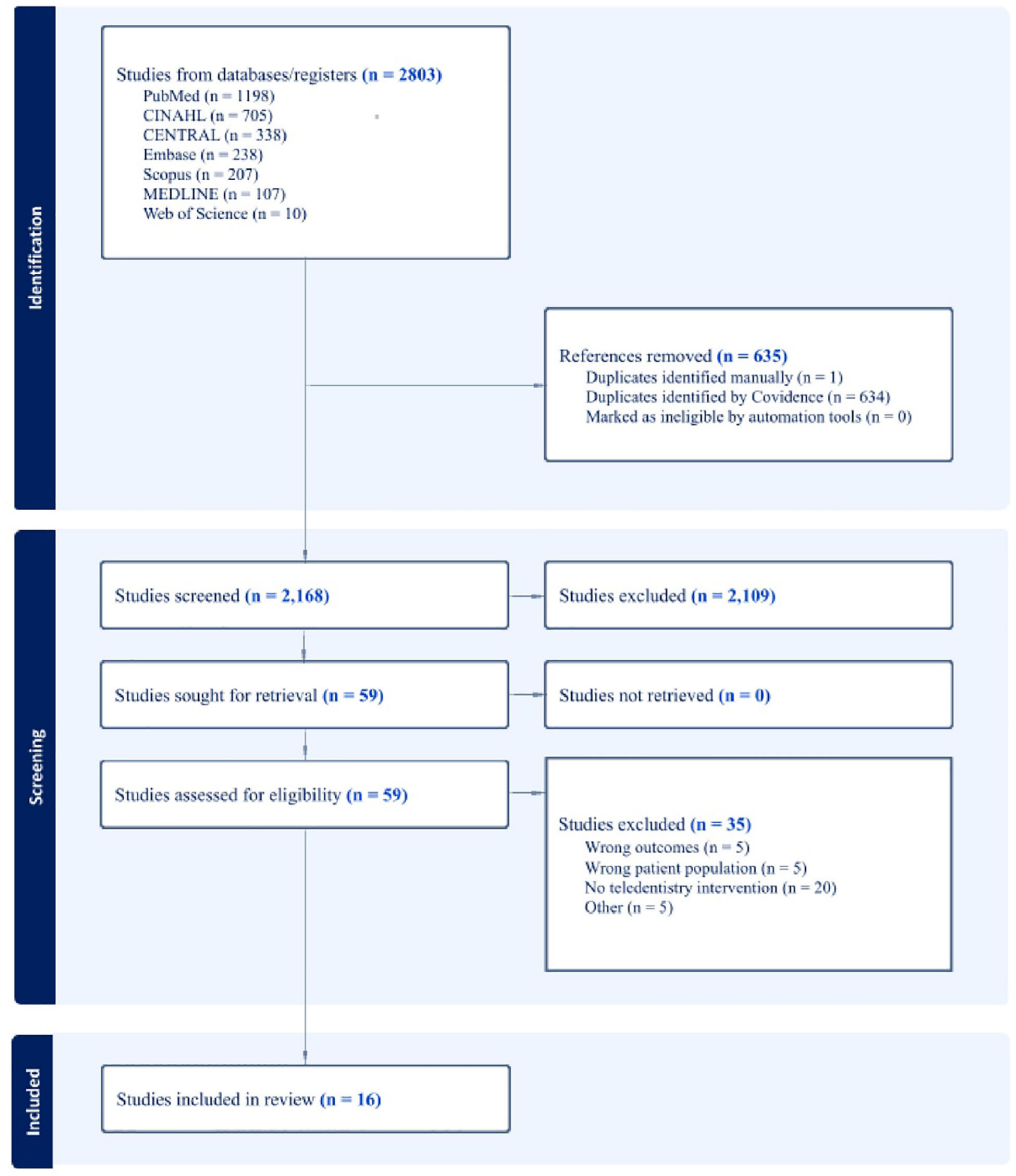
PRISMA 2020 flow diagram of the study selection process.

Following PRISMA guidelines, the initial search identified 2803 records. After removing duplicates (634 automated and one manual), 2168 studies underwent title and abstract screening, with 2117 excluded as irrelevant. Fifty‐nine full‐text articles were assessed for eligibility, of which 35 were excluded on the basis of predefined criteria. Sixteen studies met all inclusion criteria and were included in the final synthesis (Figure 1).

Sixteen intervention studies conducted between 2004 and 2024 were included (Table 2). They reported 15 distinct interventions; the difference is accounted for by Tynan et al. [20, 21], who reported on two studies testing the same intervention. The cumulative sample included 1747 older adults, with individual study sizes ranging from 4 to 252. Most studies were conducted in high‐income countries, such as the United States (*n* = 3), Japan (*n* = 3), Australia (*n* = 3), and Chile (*n* = 2). Importantly, no intervention was implemented in PHC settings, and all findings should, therefore, be interpreted as having indirect applicability.

**TABLE 2:** Overview of included studies: teledentistry interventions for oral health management in homebound and institutionalized older adults.

Study's first author [reference]	Study design	Population	Sample size (*n*)	Age (years)	Setting
Ako [22]	Observational	Institutionalized older adults	60	≥60	LTCF
Beltrán [11]	Interventional	Older adults	135	≥60	Community
Beltrán [23]	Observational	Community‐dwelling older adults	76	≥60	Community
Bradley [24]	Observational	Older adults	41	≥60^a^	Community
Estai [8]	Diagnostic study	Adults/older adults	100	Mean age reported (∼65)	Clinical
Hartshorn [5]	Program evaluation	Institutionalized older adults	39	≥60	LTCF
Kandala [25]	Diagnostic study	Institutionalized older adults	100	≥60	LTCF
Kim [26]	Interventional	Older adults	11	≥60	Home‐based
Lee [27]	Mixed‐methods	Caregivers of older adults	134	≥60^a^	LTCF
Niknam [28]	Diagnostic study	Older adults	109	≥60	Clinical
Sekiguchi [29]	Interventional	Older adults	47	≥60	Community
Silva [30]	Validation study	Institutionalized older adults	34	≥60	LTCF
Tepper [31]	Interventional	General	Not reported	Not reported^b^	Home‐based
Tomuro [32]	Interventional	Older adults	4	Not reported^a^	Home‐based
Tynan [20]	Program study	Older adults	116	≥60	LTCF
Tynan [21]	Comparative study	Older adults	252	≥60	LTCF

^a^ Age was not explicitly reported; however, the population was clearly described as older adults on the basis of the study context and inclusion criteria.

^b^ Age was not reported; intervention was applied to the general homebound population.

The interventions varied in duration, ranging from 1.9 to 17 months, with an average duration of 6.7 months (±5.1). The technological approaches used in these interventions were predominantly asynchronous (62.5%, *n* = 10), allowing for remote consultations via store‐and‐forward methods, whereas synchronous (25%, *n* = 4) and hybrid models were also employed (12.5%, *n* = 3).

The most common devices for data collection and patient assessment included smartphone‐based imaging tools (50%, *n* = 8) and intraoral cameras (37.5%, *n* = 6).

The included studies described 15 distinct teledentistry interventions, summarized in Table 3 by type, delivery model, and main outcomes.

**TABLE 3:** Summary of included teledentistry interventions.

Study's first author [reference]	Type of intervention	Delivery model	Main outcome
Ako [22]	Oral health assessment (video‐based)	Asynchronous	Feasibility of video‐based oral assessment
Lee [27]	Digital oral care support	Hybrid	Caregiver acceptance and usability
Kandala [25]	Diagnostic concordance	Asynchronous	Agreement between in‐person and TD decisions
Hartshorn [5]	Virtual dental home	Asynchronous	Improved access to preventive care
Beltrán [23]	Oral health assessment (platform)	Asynchronous	Association with geriatric conditions
Niknam [28]	AI‐assisted diagnosis	Asynchronous	High diagnostic agreement
Tepper [31]	Interprofessional telehealth	Hybrid	Improved care coordination
Beltrán [11]	Web‐based TD platform	Hybrid	Patient satisfaction
Silva [30]	Video‐based assessment validation	Asynchronous	Interobserver reliability
Kim [26]	mHealth rehabilitation	Asynchronous	Improved swallowing pressure
Tynan [20]	Integrated oral health model	Hybrid	Improved access and outcomes
Tynan [21]	Comparative program	Hybrid	Improved care pathways
Estai [8]	Caries screening	Asynchronous	High specificity
Sekiguchi [29]	Oral function rehabilitation	Synchronous	Improved swallowing and function
Bradley [24]	Referral system	Asynchronous	Efficient triage
Tomuro [32]	Telecare tutorials	Synchronous	Improved oral care behaviors

The review identified four primary categories of teledentistry use: oral health assessment, oral health education, oral function rehabilitation, and treatment planning and referral. These categories represent practices described in the literature that may have potential applicability to PHC settings.

Most studies focused on oral health assessment (50%, *n* = 8), emphasizing the use of remote diagnostic tools for early detection of oral diseases. Treatment planning and referral accounted for 37.5% (*n* = 6) and aimed to improve access to specialized care. In 25% of the studies (*n* = 4), oral health education interventions were implemented, targeting caregivers and older adults to enhance their daily oral care routines. Oral function rehabilitation, which addressed swallowing difficulties and denture‐related complications, was the least explored category (12.5%, *n* = 2).

Smartphones (50% of studies) and intraoral cameras (37.5%) were dominant technologies, primarily used in asynchronous workflows (62.5%) for rural and low‐connectivity settings. Synchronous videoconferencing (25%) supported real‐time feedback, as seen in interprofessional consultations.

Interprofessional collaboration was a key component in many studies, with dental teams working alongside nurses (*n* = 3) and dental hygienists (*n* = 3), which improved outcomes, exemplified by remote specialist input [21] with innovations like AI‐driven diagnostics [28], hybrid platforms [31], and nurse–dentist concordance (*κ* > 0.70) [30].

Clinical indicators evaluated across studies included caries experience, with *κ* values ranging from 0.57 to 0.89 in mobile teledentistry assessments [8], gingival inflammation, denture fit, swallowing tongue pressure, and soft tissue lesions [5, 11, 19]. AI‐assisted remote assessments for oral mucosal lesions achieved *κ* values above 0.81, indicating substantial agreement [5].

Patient‐centered outcomes included significant improvements in swallowing tongue pressure following mHealth interventions [26] and better oral hygiene compliance reported in telemonitoring programs [27, 32]. Implementation studies in rural Queensland described reductions in unnecessary referrals and improved care pathways with associated cost savings [14, 20, 21].

Technical challenges were frequent, with barriers such as internet connectivity in rural areas [20, 21] and camera positioning errors affecting 12% of video assessments [30]. Low‐digital literacy among patients and caregivers was also reported as a common obstacle in different studies [7, 27].

### 3.1 Oral Health Assessment

Eight studies (50%) [8, 21, 22, 23, 24, 27, 28, 30] highlighted the effectiveness of digital tools for diagnosing and monitoring oral conditions. In Chile, the TEGO platform was applied to assess oral health in a rural Mapuche community, revealing high prevalences of xerostomia (63.2%) and periodontal disease (83%), both associated with geriatric health [23]. In Norway, the SmartJournal tool was utilized in nursing homes to assess caregiver acceptance, incorporating monthly oral health tracking with e‐learning modules [27]. In Brazilian nursing homes, the ASBTO tool validated video‐based assessments, reporting moderate reliability for natural teeth (*κ* ≤ 0.12) but strong agreement for denture conditions (*κ* ≥ 0.85) [30]. A study in Japanese long‐term care facilities demonstrated the efficacy of smartphone‐recorded intraoral videos for assessing mucosal health and denture fit [32]. In Australia, live‐stream teledentistry using intraoral cameras in rural institutions reduced unnecessary referrals by 12% [20].

Two studies emphasized asynchronous systems for triaging oral medicine cases [24] and caries screening [8], with the latter achieving 97%–98% specificity for smartphone‐based caries detection. Finally, an AI‐enhanced web platform demonstrated strong diagnostic agreement for oral lesions, with *κ* values ranging from 0.81 to 0.9 [28].

### 3.2 Oral Health Education

Four studies (25%) [22, 26, 27, 32] focused on remote educational interventions. Videophone tutorials for homebound older adults in Japan improved functional independence scores by 38% [30]. Fax‐supported tutorials with coordinator‐mediated exercises in Japanese island communities enhanced mastication and swallowing [32]. A mobile health app combined with biweekly coaching increased median swallowing pressure by 51% (17.5–26.5 kPa; *p* = 0.046) in older adults with dysphagia [26]. However, e‐learning modules integrated into digital platforms faced operational challenges, such as holiday disruptions [27].

### 3.3 Oral Function Rehabilitation

Two studies (12.5%) [26, 29] addressed oral functional improvements through remote interventions. Significant gains in swallowing (via the Repetitive Saliva Swallowing Test) and articulation (oral diadochokinesis) were reported among Japanese older adults [29]. An app‐based biofeedback demonstrated effectiveness for tongue‐strengthening exercises, emphasizing its role in dysphagia treatment [26].

### 3.4 Treatment Planning and Referral

Six studies (37.5%) [5, 11, 20, 25, 29, 31] emphasized patient referral through teledentistry. A platform developed during the COVID‐19 pandemic enabled remote specialist consultations, achieving patient satisfaction rates above 75% [11]. Travel burdens in rural Australia were reduced through teledentistry referrals [20]. Asynchronous screenings in long‐term care facilities and synchronous video consultations with homebound patients were implemented [5, 31]. An 87% agreement (*κ* = 0.74) between in‐person and teledentistry treatment planning decisions for special‐needs patients was reported [25].

Clinically, teledentistry reduced unnecessary referrals by 12% [29]. A rural program produced a 40% cost savings [23] by optimizing referral pathways, underscoring teledentistry's scalability in resource‐limited settings.

## 4 Discussion

Oral health is a fundamental component of primary care, as it directly influences overall health and daily functioning. Despite being largely preventable, oral diseases remain a significant cause of morbidity worldwide and continue to be neglected, particularly in rural and under‐resourced settings. Addressing this global challenge requires policy reforms that integrate oral health into PHC, with a strong emphasis on prevention and equitable access to care.

In this context, teledentistry has emerged as a potential approach to improving access to oral healthcare for homebound and institutionalized older adults, particularly in underserved populations. This rapid review synthesizes evidence on teledentistry interventions and highlights both their potential and the challenges associated with their implementation. Importantly, none of the included studies were conducted within PHC settings, and therefore, the findings should be interpreted in terms of indirect applicability to PHC.

Overall, the findings suggest that teledentistry may help reduce access barriers and support care. Asynchronous and hybrid models demonstrate substantial diagnostic accuracy for conditions, such as dental caries [8], gingival inflammation, denture fit, swallowing tongue pressure, and soft tissue lesions [24, 26, 28, 30]. Synchronous models have also effectively optimized triage processes, reducing unnecessary specialist referrals [20]. Additionally, teledentistry has proven to be cost‐effective, with a 40% reduction in transportation costs due to optimized referral pathways [3, 5, 23].

These findings support the role of teledentistry as a complementary strategy to improve access to oral healthcare, particularly through remote diagnostic and treatment planning capabilities. By enabling earlier identification of oral conditions and reducing logistical constraints, teledentistry may enhance care delivery for homebound and institutionalized older adults. Although previous systematic reviews have highlighted the feasibility and cost‐effectiveness of teledentistry [3, 4], this study provides a more detailed analysis of implementation challenges, interprofessional collaboration, and sustainability within universal health systems.

Several studies have demonstrated a high level of agreement between teledentistry‐based assessments and in‐person diagnoses, particularly for dental caries, periodontal disease, and oral lesions [8, 28, 30]. Synchronous and hybrid models have significantly improved triage efficiency, reducing specialist referrals [20]. These findings align with systematic reviews emphasizing teledentistry's diagnostic reliability and cost‐effectiveness in resource‐limited settings [3, 4, 33, 34]. However, variability in caries detection sensitivity [8] underscores the need for standardized imaging protocols and training for non‐dental personnel, as reported in video‐based assessment studies [30].

Despite the widespread use of intraoral cameras and smartphone‐based imaging, several technical challenges hinder teledentistry's implementation. Studies report connectivity issues in rural areas [20], inconsistent camera positioning [30], and software interoperability limitations [8]. Additionally, systematic reviews identify low‐digital literacy among older adults and caregivers as a significant barrier, particularly in populations with cognitive impairments [33, 34]. Hybrid models that combine asynchronous data collection with synchronous feedback [23, 26] and user‐friendly platforms [27] have been proposed to mitigate these limitations.

Integrating oral health therapists, nurses, and remote specialists into teledentistry services has been shown to enhance care coordination and increase patient adherence to oral care plans [24, 30]. Systematic reviews emphasize the need for training programs to improve digital competencies among caregivers and standardized referral workflows [7, 9]. However, challenges persist, such as regulatory gaps in licensing remote consultations [3] and increased workload pressures on healthcare providers during emergencies [27].

Teledentistry has been recognized for expanding access. However, reviews caution that without universal access policies, teledentistry may exacerbate disparities, particularly for older adults with limited mobility or low socioeconomic status [4, 33]. Ethical concerns, including informed consent for cognitively impaired patients and data privacy and security issues, highlight the need for regulatory frameworks that balance technological advancements with patient rights [2, 9].

Integrating teledentistry into PHC aligns with global efforts to enhance equitable access to oral healthcare. Universal health systems have demonstrated that structured referral pathways and national telehealth programs can facilitate widespread adoption of teledentistry [35]. Additionally, teleeducation initiatives, including caregiver‐facing apps [27] and videophone tutorials [32], present opportunities to bridge oral health literacy gaps. Policy recommendations should prioritize reimbursement mechanisms for asynchronous consultations and digital competency training for primary care professionals to ensure sustainability [3, 9].

Although this review provides a comprehensive literature synthesis, several limitations should be noted. The included studies exhibited heterogeneity in design, sample size, and outcome measures, complicating direct comparisons. Three of them did not specify an age range over 60 years, and one study presented an intervention to the general homebound population. Additionally, most research was conducted in high‐income countries (United States, Japan, and Australia), which limits the generalizability and applicability of the findings to low‐ and middle‐income settings. Furthermore, none of the studies were implemented within PHC settings, potentially restricting the applicability of the findings to community‐based dental care models.

A critical gap remains in long‐term follow‐up data, which hampers understanding of teledentistry's sustained effects on oral health outcomes and continuity of care. Future research should prioritize longitudinal assessments of teledentistry and comparative analyses of traditional in‐person care versus teledentistry interventions for homebound and institutionalized older adults. Crucially, studies must evaluate the integration of teledentistry into PHC systems to assess its capacity to improve access, equity, and preventive care delivery in community‐based settings [34, 35, 36, 37]. These investigations will be essential to elucidate differences in clinical outcomes, patient satisfaction, and cost‐effectiveness. Additionally, further work is needed to explore implementation strategies in low‐ and middle‐income countries, focusing on overcoming infrastructure challenges, workforce training for PHC providers, and developing sustainable funding models. Integrating AI‐driven diagnostics [28] and portable intraoral scanners [20] represents a promising avenue to enhance diagnostic accuracy in resource‐limited PHC settings. Moreover, future research should evaluate the interoperability of teledentistry systems with electronic health records, as challenges related to system integration and workflow coordination have been reported in the literature [7].

By integrating oral health into broader population‐oriented healthcare frameworks, teledentistry aligns with PHC underpinnings—comprehensive, continuous, accessible, and equitable care—while demonstrating benefits in diagnostic accuracy, optimized referral pathways, and enhanced care coordination [16].

To translate digital advances into sustainable practice, a dual approach targeting both infrastructure and workforce capacity is required. National health systems must invest in reliable connectivity and affordable, user‐friendly devices, while also creating policies that recognize and reward remote consultations. Concurrently, it is essential to strengthen workforce capacity through hands‐on training in standardized teledentistry protocols for dentists, nurses, caregivers, and community workers, thereby promoting widespread digital health literacy.

Clear regulatory frameworks are also essential: Data privacy and informed‐consent procedures must be tailored to protect personal data. Engaging technology partners to develop interoperable, age‐appropriate platforms—integrated with existing electronic health records and supported by transparent, bias‐mitigated decision‐support algorithms—will streamline workflows and improve the experience of older patients and their caregivers.

Looking ahead, rigorous longitudinal and comparative studies are needed to assess long‐term clinical outcomes, cost‐effectiveness, and equity impacts across diverse settings, including low‐ and middle‐income countries. Evaluations should examine not only oral indicators, such as caries progression and periodontal health, but also patient autonomy, caregiver burden, and the social determinants that influence oral care uptake and access. By combining oral health policy frameworks, professional training, and science‐based technological innovation, teledentistry may evolve from a promising concept into a supportive strategy within PHC, reducing barriers related to geography, mobility, and socioeconomic conditions.

### 4.1 Indirect Applicability to PHC

None of the included studies were conducted within PHC settings. Therefore, the findings of this review should be interpreted as evidence of indirect applicability rather than direct implementation.

The teledentistry practices identified—such as oral health assessment, education, rehabilitation, and referral—were implemented in contexts, including long‐term care facilities, home‐based settings, and specialized services. Although these interventions demonstrated feasibility and potential effectiveness, their translation into PHC requires careful consideration of contextual factors, including workforce organization, infrastructure, and integration with existing care pathways.

Thus, the findings of this review provide a conceptual and practical basis for informing the future implementation of teledentistry within PHC. However, successful integration depends on alignment with core PHC principles, including access, continuity, and care coordination [13], as well as broader health system strategies that emphasize integrating oral health into primary care [12, 16]. Further studies are needed to evaluate the feasibility, effectiveness, and real‐world implementation in PHC settings.

## 5 Conclusions

Teledentistry shows potential to improve access to oral healthcare and support clinical decision‐making for homebound and institutionalized older adults. The findings of this review suggest that a range of teledentistry practices may be applicable to PHC; however, as none of the included studies were conducted within PHC settings, conclusions regarding implementation should be interpreted with caution.

Reported challenges, including technical barriers and digital literacy limitations, indicate that successful integration into PHC is likely to require investments in connectivity, professional training, and standardized protocols. Future studies should evaluate the feasibility, effectiveness, and integration of teledentistry within PHC contexts.

## Author Contributions

Conceptualization: Gabriel Schmitt da Cruz and Ana Lúcia Schaefer Ferreira de Mello. Methodology: Gabriel Schmitt da Cruz, Gabriela Bampi, Eduardo Dickie de Castilhos, Maria Inês Meurer, Jose Antonio Gil‐Montoya, and Ana Lúcia Schaefer Ferreira de Mello. Software: Gabriel Schmitt da Cruz and Elaine Caroline Ferreira. Data curation: Gabriel Schmitt da Cruz and Elaine Caroline Ferreira. Investigation: Gabriel Schmitt da Cruz, Elaine Caroline Ferreira, Gabriela Bampi, Eduardo Dickie de Castilhos, Maria Inês Meurer, Jose Antonio Gil‐Montoya, and Ana Lúcia Schaefer Ferreira de Mello. Validation: Gabriel Schmitt da Cruz and Ana Lúcia Schaefer Ferreira de Mello. Formal analysis: Gabriel Schmitt da Cruz, Elaine Caroline Ferreira, and Gabriela Bampi. Supervision: Eduardo Dickie de Castilhos and Ana Lúcia Schaefer Ferreira de Mello. Funding acquisition: Ana Lúcia Schaefer Ferreira de Mello. Project administration: Ana Lúcia Schaefer Ferreira de Mello. Writing – original draft: Gabriel Schmitt da Cruz, Elaine Caroline Ferreira, Gabriela Bampi, Eduardo Dickie de Castilhos, Maria Inês Meurer, Jose Antonio Gil‐Montoya, and Ana Lúcia Schaefer Ferreira de Mello. Writing – review and editing: Gabriel Schmitt da Cruz, Eduardo Dickie de Castilhos, Maria Inês Meurer, Jose Antonio Gil‐Montoya, and Ana Lúcia Schaefer Ferreira de Mello.

## Funding

This study was supported by (Grant Number—402673/2023‐9).

## Ethics Statement

The authors have nothing to report.

## Conflicts of Interest

The authors declare no conflicts of interest.

## Data Availability

The data supporting the findings of this study are available from the corresponding author upon reasonable request.
